# Update on the Benefits and Mechanisms of Action of the Bioactive Vegetal Alkaloid Berberine on Lipid Metabolism and Homeostasis

**DOI:** 10.1155/2018/7173920

**Published:** 2018-07-02

**Authors:** Yanwen Wang, Jeffrey A. Zidichouski

**Affiliations:** ^1^National Research Council of Canada, 550 University Avenue, Charlottetown, PE, Canada C1A 4P3; ^2^Biomedical Sciences, University of Prince Edward Island, 550 University Avenue, Charlottetown, PE, Canada C1A 4P3; ^3^Industrial Research Assistance Program, National Research Council of Canada, Calgary, AB, Canada T2L 2K7; ^4^Physiology and Pharmacology, University of Calgary, Cummings School of Medicine, Calgary, AB, Canada T2N 1N4

## Abstract

Elevation of circulating levels of blood cholesterol, especially LDL cholesterol, and/or the decrease of HDL cholesterol levels have long been recognized as primary risk factors for developing atherosclerosis that leads to cardiovascular and cerebrovascular disease. Hypertriglyceridemia is an independent risk factor that is known to contribute to the development of atherosclerosis. Thus, various interventional efforts aimed at reducing hypercholesterolemia and hypertriglyceridemia have been practiced clinically for decades to reduce morbidity and mortality risk associated with deleterious cardiovascular and cerebrovascular events. As such, many drugs have been developed and clinically used to treat hypocholesteremia and/or hypertriglyceridemia; however, dietary approaches including supplements along with changes in nutrition and lifestyle have become increasingly attractive and acceptable methods used to control borderline or moderately increased levels of blood cholesterol and triacylglycerols. In this regard, the use of a plant/herbal bioactive compound, berberine (BBR), has recently been studied extensively in terms of its efficacy as well as its mechanisms of action and safety as an alternative intervention that beneficially modulates blood lipids. The aim of this review is to provide a comprehensive update on BBR research, new concepts and directions in terms of product development and current challenges, and future prospects of using BBR to manage diseases and complications associated with dyslipidemia.

## 1. Background 

**Figure 1:**
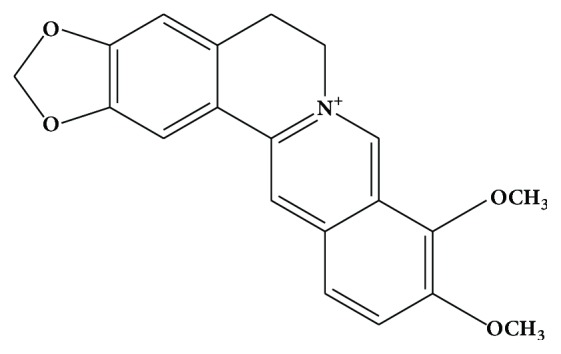
Structure of berberine.

Berberine (BBR) is a quaternary ammonium salt derived from the protoberberine group of benzylisoquinoline alkaloids (Figure 1). It is the principal bioactive compound found in* Coptis chinensis* (Chinese goldthread),* Hydrastis canadensis* (goldenseal),* Berberis vulgaris *(barberry), and many other medicinal plants [1, 2]. It has many chemical forms such as BBR hydrochloride, BBR sulfate, BBR citrate, and BBR phosphate [3]. There are a number of commercial suppliers of pure BBR and concentrated plant extracts that contain significant amounts of BBR. The majority of BBR suppliers are located in China and followed by India. Each of the suppliers provide product specifications, raw material source, extraction method, and analytical information regarding product purity and chemical properties. BBR chloride is the predominant salt form of BBR and has a purity of 97% or higher.

Information from historical use and modern scientific research indicates that BBR possesses a wide range of biological and pharmacological effects. It has been used for thousands of years in traditional Chinese medicine to treat various diseases, such as infectious diseases and gastrointestinal disorders, without apparent side effects being reported from traditional use or from clinical use [4, 5]. The cholesterol-lowering effect of BBR was first reported in 1989 with the observation that BBR lowered intracellular cholesterol in cultured human aortic intimal cells [6]. However, the potential health benefits escaped serious attention and it was not until 2004 when BBR was found to markedly lower blood cholesterol levels* in vivo* in both human subjects and hamsters [7]. Since then, a large number of studies have confirmed the benefits of BBR on cholesterol homeostasis by lowering total cholesterol (T-C), LDL cholesterol (LDL-C), non-HDL cholesterol (non-HDL-C) [8–10], or triacylglycerols (TAG) [11–13], and further studies have revealed that several mechanisms are involved in the regulation of cholesterol metabolism by BBR [10, 14]. This review is intended to provide an update on recent advances made in BBR research and to discuss the potential use of BBR in the development of new products for maintaining health lipid profile or reducing hypercholesterolemia and hypertriglyceridemia. In addition, the mechanisms of action, bioavailability, and safety of BBR are also examined and discussed regarding its use as a natural lipid-lowering agent.

## 2. Dose, Administration Route, and Efficacy of BBR on Lipid Reduction in Animals

BBR has been studied in several different animal models for its cholesterol- and triacylglycerol-lowering effects, including models of high-cholesterol diet-induced hypercholesterolemia, high-fat diet-induced hyperlipidemia, high-fat and high-carbohydrate (sugar) diet-induced hyperlipidemia, genetic models for hyperlipidemia and diabetes, and drug- (streptozotocin and alloxan) induced diabetes (Table 1). Various animal species have been used in the BBR studies, which include rats, mice, and hamsters, with the majority of the experiments being conducted on male rodents and only a few studies have been carried out on female rodents or in experimental groups containing both sexes. BBR was administered through different routes, including intragastric injection or oral gavage feeding (i.g.), intraperitoneal injection (i.p.), and dietary supplementation. Dose levels ranged from 15 mg/kg to 562.5 mg/kg per day i.g. or dietary supplementation and from 1.5 mg/kg to 5 mg/kg per day when administered i.p..

### 2.1. Cholesterol-Lowering Effect of BBR in Diet-Induced Hypercholesterolemia in Rodents

#### 2.1.1. BBR Lowers Blood Cholesterol through Oral Administration

There are several different ways to induce hypercholesterolemia in rodents (Table 1) but using diet-induced hypercholesterolemia best mimics the human condition. The reason for this is that humans develop hypercholesterolemia predominately as a result of poor dietary habits and poor lifestyle choices. In the majority of studies, BBR was administered through gavage feeding [10, 19] while in a few studies i.p. injection [9, 15] or dietary supplementation [11] was employed. Studies using the listed models in Table 1 have shown significant effects of lowering blood T-C, LDL-C, and/or non-HDL-C. It is noted that different efficacies of BBR were reported, which might be a result of differences in diet, BBR dose level, treatment time, animal species or strain, animal health and disease condition, and experimental design (e.g., prevention versus treatment studies).

Golden Syrian hamster has long been considered as a good rodent model for studying human cholesterol metabolism and homeostasis [69]. BBR studies using this animal model have consistently resulted in significant reductions of T-C, non-HDL-C, and LDL-C when examined using a number of different experimental designs. In high-cholesterol diet-induced hypercholesterolemic hamsters, 100 mg/kg·d of BBR for 10 days following a 2-week induction period of hypercholesterolemia resulted in reductions of 27% of blood T-C and 39% of LDL-C [33]. In another study, hamsters with hypercholesterolemia induced by a high-fat and high-fructose diet for 4 weeks, a 2-week treatment of BBR at 150 mg/kg·d resulted in a 35% reduction of blood T-C [18]. In female C57BL/6J mice that were pretreated with BBR at 10 or 30 mg/kg·d for 4 weeks and then fed a high-cholesterol diet for 7 days, dose-dependent reductions of 43% (10 mg/kg·d) and 57% (30 mg/kg·d) in T-C, 48% and 61% in LDL-C, and increase of 38% and 70% in HDL-C were observed, respectively, as compared to unpretreated mice [24]. Similar effects of BBR were reported from other studies conducted on male [17, 19, 24] and female [21, 24, 25] rats, mice [15, 24], and Golden Syrian hamsters [7, 13] using diet-induced hypercholesterolemia. A further study reported that in male SD rats subjected to a high-fat and high-cholesterol diet administration of BBR at doses of 50, 100, and 150 mg/kg·d for 8 weeks reduced T-C by 29-33% and non-HDL-C by 31-41%, respectively, with no significant differences observed between the three different doses [10]. BBR treatment appears to be quite effective in preventing and treating hypercholesterolemia. Although 100 mg/kg·d BBR was used in most studies, the EC_50_ dose may be found to be substantially lower, such as below 50 mg/kg·d [10] or 10 mg/kg·d [24] or even lower.

#### 2.1.2. BBR Lowers Blood Cholesterol via Intraperitoneal Injection

There are several studies demonstrating that i.p. injection of BBR reduced blood cholesterol to a similar extent as compared to studies that used oral gavage; however, the effective i.p. doses were 10–100-fold lower [15, 31]. For example, in male Golden Syrian hamsters fed a high-cholesterol diet, i.p. injection of BBR in a pure form or a goldenseal extract providing 1.8 mg/kg·d for 24 days reduced T-C by 32% and 30% and LDL-C by 26 and 27%, respectively [31]. Similar effects were observed in C57BL/6J mice where i.p. injection of 0.75, 1.5, and 3 mg/kg·d of BBR for 36 days reduced blood T-C by 19%, 22%, and 28%, respectively [15]. The reduction of blood cholesterol by BBR through i.p. injection was also observed in rats with hyperhomocysteinemia [23] and C57BLKS/J-Lepr^*db*/*db*^ mice [9]. Although similar cholesterol-lowering efficacies can be achieved at a much lower dose when BBR is administrated intraperitoneally, i.p. injection is not a suitable route of administration for use in humans.

### 2.2. BBR Lowers Blood Cholesterol in Diabetic Rodents

The cholesterol-lowering effect of BBR has been observed not only in different hyperlipidemic rodent models but also in animals with diabetes or insulin resistance (Table 1). In accordance with the observations in hypercholesterolemic rodents, many antidiabetic studies showed significant effects of BBR on blood cholesterol reduction. As hyperlipidemia is a complication of diabetes, reduction of cholesterol is beneficial to diabetic patients in addition to the glucose-lowering benefits. In male Kunming mice with streptozotocin- (STZ-) induced diabetes and with free access to a regular chow, oral administration of BBR at 100 mg/kg·d for 4 weeks reduced blood T-C by 16% and LDL-C by 20% while increasing HDL-C by 9% [16]. A stronger effect was observed in male diabetic KKAy mice fed a high-fat diet [12]. In this study, oral gavage of BBR at 250 mg/kg·d once a day for 4 weeks reduced blood T-C by 42%. As the treatment period and administration route were the same, the difference of cholesterol-lowering efficacy between these two studies appeared to be a result of the differences in blood cholesterol baseline and dose of BBR. A higher baseline blood T-C concentration was seen in KKAy mice [12] than in Kunming mice [16], providing a larger room for reduction in response to BBR treatment. Similar effects were reported in rats with STZ-induced diabetes and free access to a high-fat diet [26, 28] and Golden Syrian hamsters with STZ-induced diabetes and free access to a high-fat and high-cholesterol diet [21]. In accordance with findings in hypercholesterolemic rodents, the dose 100 mg/kg·d appeared to be the most effective in diabetic or insulin resistant animals [17, 32], and further increases of BBR dose did not produce better effects [30, 34]. The majority of the studies were carried out in STZ-induced diabetic models, and amongst these studies, some were provided with a high-fat diet while others were fed a regular diet, indicating that BBR lowers blood cholesterol in diabetic animals independent of the diet. While studies were conducted predominantly in male rodents, a few experiments were carried out on females [24, 25]. The cholesterol-lowering effect of BBR appeared to be independent of gender and stronger effects were seen in animals that had higher baseline cholesterol levels.

In summary, a number of studies have been conducted in diet-induced hypercholesterolemic and diabetic rodents. Different types of diets were used to induce hypercholesterolemia, which include (1) high-cholesterol diet, (2) high-fat diet, (3) high-fat and high-cholesterol diet, and (4) high-fat and high-carbohydrate diet. BBR is normally administered through diet or oral gavage, and in a few studies i.p. injection was used. It is understandable based on bioavailability (see Bioavailability) that dose levels used in i.p. injection were much lower than those used via dietary supplementation or oral gavage. However, whether by i.g., diet, or i.p. administration, BBR doses used varied significantly. As well, the durations of BBR treatment were largely different. Based on the current literature, the optimal dose of BBR given orally is not more than 100 mg/kg·d and no further benefit can be achieved at a dose level over 100 mg/kg·d. As BBR doses of 10–100 mg/kg·d showed significant effects and good efficacies in different studies, further studies are required to optimize the dose. In addition, an effectively lower dose could be achieved through product innovation such as emulsion and nanotechnology aimed at improving BBR bioavailability. Although i.p. injection of BBR showed significant effects, this route of administration does not have an attraction of application due to its invasiveness and inconvenience. Another aspect that should be noted is the treatment duration where generally a minimum treatment time of 4 weeks for hamsters and 6 weeks for rats or mice should be employed to achieve a stable cholesterol-lowering effect of a treatment or agent.

## 3. Lipid-Lowering Efficacy of BBR in Humans

With a large body of evidence demonstrating the beneficial effect of BBR on the regulation of cholesterol metabolism and homeostasis in the preclinical studies, clinical trials have been carried out to validate the effects of BBR in human subjects (Table 2). To date, a number of human trials have been performed on subjects with different health and disease conditions including those with hypercholesterolemia [7, 53, 55], diabetes [41, 42], metabolic syndrome [46, 51], postmenopause [52], and cardiovascular disease [44], as well as subjects that were hypercholesterolemic and took statin drugs [48]. The majority of the human trials showed reductions of 11–29% in T-C and 8–25% in LDL-C while a few did not show significant effects [42, 46]. The observed variations in the cholesterol-lowering efficacy of BBR might be a result of confounding effects of differences in experimental design, subject condition, treatment period, dose, and frequency of BBR administration. In terms of experimental design, at least single arm, parallel arm, and crossover studies were used. Further, these clinical trials used BBR in a broad range of doses and employed different administration frequencies that ranged from 0.5 to 1.5 g/d and were administered once, twice, or thrice daily. Taken all together, it is apparent that both the dose and frequency of BBR administration need to be further examined and optimized, especially through well-designed random controlled clinical trials.

Pure BBR was used in most of the clinical studies [7, 36, 42], while plant/herbal extracts with equivalent amounts of BBR were used in other trials [31, 50]. Both pure BBR and BBR-enriched extracts showed significant effect of lowering blood cholesterol. The treatment period ranged from 4 weeks to 12 months [49, 53]. Interestingly, there appears to be a BBR plateau effect as long-term studies for either 12 months [53] or 6 months [48] did show similar efficacies in terms of cholesterol lowering as compared to the effects reported from shorter-term studies of only 4 weeks [7, 36]. These finding are consistent with the notion that the BBR effect plateaus and remains stable over a relatively long treatment time. In general, it appears that a minimum of 4 weeks of treatment is required in humans for the determination of stable lipid-lowering efficacy of a product [70, 71]. In most studies, dietary restrictions were not applied or well controlled, e.g., the patients or participants maintained their normal dietary habits during the course of the studies. Furthermore, the BBR-induced effect was found to be significant and stable in subjects with different metabolic phenotypes such as hypercholesterolemia [7, 53], cardiovascular disease [44], diabetes [41, 42], metabolic syndrome [51], cancer, or polycystic ovary syndrome [54].

It should be pointed out that in some clinical studies BBR was administered in a tablet form of a nutraceutical product [47, 48], comprised of 500 mg BBR, 200 mg red yeast extract, 10 mg policosanol, 0.2 mg folic acid, 2 mg CoQ_10_, and 0.5 mg astaxanthin. This product has consistently shown a marked cholesterol-lowering effect. For instance, in hypercholesterolemic subjects, the combination reduced T-C by 17% and LDL-C by 23% [55]. The cholesterol-lowering effect of this combination product was also investigated in elderly (>75 y) hypercholesterolemic patients who were intolerant to statins and in this subgroup the product resulted in reductions of 20% in T-C and 31% in LDL-C [53]. In hypercholesterolemic patients who were intolerant or refused to take statins, the combination lowered T-C by 24% and LDL-C by 32%, which was more effective than the cholesterol absorption inhibitor drug ezetimibe that showed 19% and 25% reductions of T-C and LDL-C, respectively [48]. A comparison has been conducted between BBR at 0.5 g/d and a nutraceutical combination that contains 0.5 g of BBR. The results demonstrated that 0.5 g/d of BBR showed a similar efficacy with the combination product, suggesting that BBR is the primary cholesterol-lowering component of the product [49]. In a recent study, a second nutraceutical product that contains 200 mg BBR, 3 mg monacolin K, 10 mg chitosan, and 10 mg CoQ_10_ once a day for 12 weeks reduced LDL-C by 19% and non-HDL-C by 15% [37]. It is not clear that the lower cholesterol efficacy of this product was a result of lower dose of BBR or confounded by effects of BBR interaction with other components in this particular formulation [49].

Several systematic reviews and meta-analyses of randomized controlled trials have consistently demonstrated the beneficial effects of BBR or BBR-containing nutraceuticals on blood T-C, LDL-C, and HDL-C [72–74]. It was concluded in a meta-analysis that the reduction of blood cholesterol obtained with BBR supplementation is similar to or comparable with that produced by statin therapy [75]. More recent reviews showed that BBR alone and in combination with other dietary supplements provided an average LDL percentage lowering capacity of 20%–30% [76, 77], while moderate intensity statin medications have been proven to lower LDL-C by 30–50% and high-intensity statins lower LDL-C even further, upward of 50% [78]. The considerable individual variations in the response of plasma cholesterol to BBR supplementation might be related to the baseline T-C and LDL-C levels [38], which are associated with genetic variants [79]. Greater reductions were seen in subjects who had higher baseline cholesterol concentrations [7, 38], in agreement with other nutraceutical products [80]. BBR represents an effective alternative for patients who are intolerant or refuse to utilize statin therapy or other drugs [74] as a matter of personal choice and could be recommended to patients with mild hypercholesterolemia or metabolic syndrome [81].

## 4. Mechanisms of Action of BBR on Cholesterol Metabolism

It is well-established that cholesterol homeostasis is determined by multiple factors and biological processes, one of which is LDL-receptor-mediated LDL cholesterol clearance in the liver [82]. Since the first report published in 2004 showing that BBR lowered cholesterol through LDL-receptor-mediated liver LDL cholesterol clearance [7], a number of studies have been conducted* in vitro* and* in vivo *to explore this particular mechanism of action [10, 83]. Many studies have shown that BBR upregulates liver LDL-receptor expression [29, 84]. Further studies suggest that BBR increases LDL-receptor expression in hepatocytes by stabilizing LDL-receptor mRNA through activation of extracellular-signal-regulated kinase (ERK) pathway [7, 85]. Other studies indicate that BBR promotes LDL-receptor expression through the proprotein convertase subtilisin/kexin type 9 (PCSK9)-LDL-receptor pathway [24, 86]. PCSK9 in hepatocytes diverts cell surface LDL-receptor towards lysosomal degradation and BBR suppresses PCSK9 expression [87]. Furthermore, BBR accelerates the degradation of hepatocyte nuclear factor 1*α* (HNF 1*α*) protein that mediates PCSK9 gene transcription [87, 88]. In accordance with these observations, changes in PCSK9 expression were correlated positively with changes of circulating LDL-C [89, 90]. The decrease in the expression of PCSK9 might also be dependent on the ERK pathway [91]. A recent study indicated that BBR promoted cholesterol excretion from liver into bile in hyperlipidemic hamsters [92], which supports the enhancement of BBR on LDL-receptor-mediated liver cholesterol clearance. More studies are required to establish relationships between ERK, HNF 1*α*, PCSK9, and LDL-receptor expression and a complete pathway through which BBR upregulates LDL-receptor-mediated liver cholesterol clearance.

In addition to LDL-receptor-mediated LDL cholesterol clearance in the liver, other critical factors such as the intestinal absorption, biosynthesis, secretion, bile acid synthesis, and secretion are also known to be involved in cholesterol homeostasis. Intestinal absorption of dietary cholesterol and reabsorption of biliary cholesterol as part of the enterohepatic circulation play a critical role in cholesterol metabolism [8, 93]. The absorption of cholesterol is controlled by multiple processes in the small intestine, including cholesterol micellarization in the intestinal lumen [94], the expression of sterol transporters [95, 96], cholesterol uptake, and the expression and activity of enzymes that catalyze the esterification of free cholesterol in enterocytes [97]. A recent study characterized the effects of BBR on these various processes and demonstrated that BBR inhibited intestinal dietary cholesterol absorption [10]. Further experiments by the same group have shown that BBR interferes with intraluminal cholesterol micellarization and decreases enterocyte cholesterol uptake* in vitro *in Caco-2 cells and permeability through Caco-2 tight junction monolayer. BBR also inhibits the gene and protein expression of acyl-coenzyme A cholesterol acyltransferase-2* in vivo* in the small intestine of rats and* in vitro* in Caco-2 cells [10]. The inhibition of acyl-coenzyme A cholesterol acyltransferase-2 expression results in a decrease of cholesterol esterification in the enterocytes and ultimately a reduction of intestinal cholesterol absorption [98].

Cholesterol clearance in the liver is achieved through the conversion of cholesterol into bile acids and/or secreted as free cholesterol in bile. It is demonstrated that BBR increases cholesterol excretion from the liver into the bile and is eliminated via the feces [92]. BBR supplementation alters bile acid profile by increasing primary bile acids while decreasing secondary bile acids in the liver and serum [99]. Further, examination of gene expression in liver tissue samples derived from hamsters treated with BBR reported an increased hepatic expression of mitochondrial sterol 27-hydroxylase [8], an enzyme that regulates bile acid synthesis from cholesterol [100]. BBR also activates cholesterol 7 alpha-hydroxylase expression and catalytic activity which also regulates bile acid synthesis [84, 101]. Taken all together, BBR enhances both cholesterol catabolism and bile acid excretion, resulting in the reductions of blood T-C and LDL-C [8, 92].

Cholesterol biosynthesis is critical to cholesterol homeostasis in which HMG-CoA reductase is well known to be the rate-limiting enzyme. It is reported that BBR inhibits the expression of this enzyme [84]. An* in vitro *study has shown that BBR inhibits cholesterol biosynthesis in hepatocytes through activation of AMP kinase (AMPK) [33]. In this study, BBR inhibited cholesterol biosynthesis in a similar manner to the AMPK activator 5-aminoimidazole-4-carboxamide 1-beta-ribofuranoside (AICAR). Activation of AMPK by BBR has also been reported in a series of diabetic studies [22, 102, 103]. Once activated, AMPK phosphorylates its downstream substrates leading to reduced ATP-consuming anabolic pathways that include cholesterol synthesis. Phosphorylation of HMG-CoA reductase by AMPK results in inactivation or reduction of cholesterol synthesis [104]. Additional support for this mechanism of action is that metformin activates AMPK [105, 106] and another study reported that the lipid-lowering effect of metformin was dependent on AMPK activation [107].

It seems that BBR regulates cholesterol metabolism through multiple mechanisms involving the intestinal dietary cholesterol absorption and biliary cholesterol reabsorption, cholesterol biosynthesis, LDL-receptor-mediated LDL cholesterol clearance, bile acid synthesis, cholesterol catabolism, excretion, cholesterol secretion into bile, and final elimination via the feces. These multiple mechanisms govern cholesterol metabolism and many of which are interregulated and involve complex positive and negative feedback loops. For example, cholesterol absorption changes reciprocally with the biosynthesis [8, 108]. Indeed, BBR inhibited intestinal dietary cholesterol absorption and reciprocally increased liver cholesterol biosynthesis in rats as measured using well-established stable isotope tracer methodologies and analysis of the isotope enrichments in cholesterol and plasma water [10]. However, there are discrepancies, which might be a result of different experimental models,* in vitro *single cell model [33, 107] that showed an inhibitory effect of BBR on cholesterol biosynthesis compared with an* in vivo *biology system that showed an upregulation of cholesterol biosynthesis while inhibiting intestinal absorption [8, 10]. The difference may suggest that BBR inhibits cholesterol biosynthesis in isolated hepatocytes* in vitro*, whereas when BBR is administered to a more complex biological system, its effect on cholesterol synthesis is abolished as a result of the strong effect on the intestinal absorption of dietary cholesterol and reabsorption of biliary cholesterol, which reciprocally and passively increases the rate of cholesterol biosynthesis as a compensatory response. However, the overall effect is that circulating cholesterol levels are still balanced negatively, leading to the overall reductions of T-C and LDL-C. The contradictory results have also ascertained that the mechanisms determined using less complex* in vitro* models or cell-based models may be different from those reported from* in vivo* models that involve more complete and complex biological systems.

## 5. Effect of BBR on Blood Triacylglycerols in Humans and Animals

Elevation of blood TAG levels is an independent contributor to atherosclerosis and cardiovascular disease. As a key component of lipid panel, TAG was analyzed in almost every study that examined the cholesterol-lowering effect of BBR in animal models (Table 1) and human subjects (Table 2). Therefore, the experimental design and conditions, including animal model, species, diet, treatment duration, administration route, and administration frequency are the same as in the cholesterol-lowering studies discussed earlier. Again, diet-induced dyslipidemia in animal is more relevant to the human etiology of hypertriglyceridemia. In this regard, high-cholesterol, high-fat, high-cholesterol and high-fat, and high-fat and high-sugar diets are used to induce hypercholesterolemia or hyperlipidemia in normal or diabetic rats, mice, and hamsters, respectively, paralleling with BBR treatment or prior to BBR treatment [7, 10, 28, 31]. In male SD rats with hypercholesterolemia induced by a high-fat and high-cholesterol diet, 100 mg/kg·d of BBR once a day for 6 weeks reduced blood TAG by 31% [11]. In another study conducted on male Golden Syrian hamsters with hypercholesterolemia induced by a high-cholesterol diet, i.p. injection once a day of pure BBR or equivalent amount of BBR in a goldenseal extract for 24 days reduced blood TAG by 33% or 34% and liver TAG by 46% or 28% [31]. A similar effect was reported in male C57BL/6J mice fed a high-fat diet, where the i.p. injection of 0.75, 1.5, or 3 mg/kg·d once daily for 36 days reduced plasma TAG by 25–38%, without differences between the doses being observed [15]. It is generally accepted that in rodents a high-fat diet induces hypotriglyceridemia instead of hypertriglyceridemia [10]. This may explain the findings that in most of the studies, BBR did not cause a significant effect on blood TAG levels in high-fat [19, 20], high-fat and high-cholesterol [10, 29, 35], or high-cholesterol [32] diet-induced hypercholesterolemia or dyslipidemia. High content of dietary carbohydrates usually results in an increase of blood TAG levels and accordingly a better reduction was seen after BBR treatment. For example, treatment with BBR at 150 mg/kg·d for 2 weeks in Golden Syrian hamsters fed a high-fat/high-fructose diet showed a 47% decrease in blood TAG and a 30% decrease in liver TAG [18]. Increased blood TAG levels are a common phenotype of diabetes, and thus reductions of blood TAG by BBR are observed more consistently in diabetic animals independent of the diet. In male Wistar rats with STZ-induced diabetes and free access to regular chow, oral administration of BBR at 187.5 mg/kg or 562.5 mg/kg once a day for 8 weeks reduced blood TAG by 66-67% [34]. After 4 weeks of i.g. supplementation of BBR at 380 mg/kg·d once a day in male Wistar rats with a high-fat diet and STZ-induced diabetes, blood TAG was reduced by 30% [30]. A similar effect was observed in male diabetic KKAy mice with free access to a high-fat diet where a reduction of 42% was produced after 4 weeks of 250 mg/kg·d i.g. supplementation once a day of BBR [12] and the same reduction was observed in STZ-induced diabetic male Wistar rats with free access to a high-fat and high-sucrose diet after 6 weeks of i.g. administration of BBR at 30 mg/kg·d once a day [28]. In STZ-induced diabetic male SD rats, i.g. injection of BBR at 100 mg/kg·d once a day for 8 weeks reduced serum TAG by 30% [26]. Several other studies in diabetic rodents showed significant reductions of blood TAG levels by BBR [16, 17, 21]. Even in rats fed a regular chow, blood TAG decreased by 35% after 2 weeks of BBR at 385 mg/kg·d once a day [25]. Evidence from a number of studies has consistently demonstrated a strong effect of BBR supplementation on blood and liver TAG levels in animals with high blood TAG levels although the efficacies are varied from one study to another.

The positive and significant effects of BBR on blood and liver TAG levels led to a number of recent studies performed on human subjects. The majority of these studies showed a consistent TAG-lowering effect of BBR (see Table 2). In subjects with metabolic syndrome or dyslipidemia, BBR reduced blood TAG by 17–22% [49, 52, 54]. In Caucasians with low levels of cardiovascular risk, BBR at 1 g/d, twice daily for 3 months, reduced TAG by 21% [44]. In Chinese patients with type 2 diabetes and dyslipidemia, 1 g/d of BBR twice a day for 3 months lowered blood TAG 36% [43]. Again, in type 2 diabetic Chinese patients, BBR at 1 g/d twice daily for 2 months or 1.5 g/d thrice daily for 13 weeks lowered TAG by 18% and 21%, respectively [41, 42]. A reduction of 21% in blood TAG was seen in type 2 diabetic patients after 8–12 weeks of treatment with BBR at 0.5–1.5 g/d [109] and also in patients with metabolic syndrome after BBR treatment at 0.9 g/d for 3 months [39]. In hypercholesterolemic subjects, blood TAG was decreased by 22% after 1 g/d of BBR for 2 months [40] or by 28% after 1 g/d for 3 months [7]. An even better effect was observed in patients with metabolic syndrome, where BBR at 1.5 g/d for 3 months reduced TAG by 42% [46]. Apparently, consistent effects are reported by different research groups in normal subjects and hypercholesterolemic, dyslipidemic, and/or diabetic patients treated with different doses of BBR for different periods of time. The majority of the studies have shown a reduction of blood TAG ranging from 17% to 22%, while high reductions (28%–42%) are seen in a few studies in patients who are diabetic and have dyslipidemia or metabolic syndrome. This notion is partially in agreement with the meta-analysis of randomized controlled trails, which concluded that BBR at a dose range of 0.6–1.5 g/d lowered blood TAG concentration from a respectable 27% up to a maximum reduction of 61% [77, 78].

## 6. Mechanisms of Action of BBR on Triacylglycerol Metabolism

The TAG-lowering effect of BBR is mediated at least in part by upregulating lipolysis gene expression and downregulating lipogenesis gene expression through AMPK signaling pathway [110]. In HepG2 cells, BBR increased AMPK activation and inhibited gene expressions of acetyl CoA carboxylase (ACC), fatty acids synthase (FAS), and glycerol-3-phosphate acyltransferase while enhancing the gene expression of medium chain acyl-CoA dehydrogenase. In different animal models and cell lines, phosphorylation and activation of AMPK are linked to the hypolipidemic effect of BBR [111, 112]. When cultured cells are exposed to an AMPK inhibitor compound C, the effect of BBR is abolished, suggesting that AMPK is a crucial player of BBR in dissipating stored fat and lower blood TAG levels [9, 113]. Further studies suggest that BBR activates AMPK by inhibiting glucose oxidation in mitochondria, resulting in an increase of AMP/ATP ratio in cells and activation of AMPK [111, 114, 115]. Activation of AMPK leads to increased fatty acid oxidation and reciprocal inhibition of lipogenesis [9]. The effects of BBR on hepatic sterol regulatory element-binding proteins (SREBPs), liver X receptor *α* (LXR*α*), and peroxisome proliferator-activated receptor *α* (PPAR*α*) transcriptional programs provide further support to the notion that BBR prevents dyslipidemia by promoting AMPK activation [116]. AMPK phosphorylates SREBP1c and SREBP2 and the phosphorylation of SREBP1c inhibits the proteolytic cleavage and nuclear translocation of SREBP1c in hepatocytes, thereby preventing its autoregulation and transcription of target lipogenesis genes ACC1, FAS, and stearoyl CoA desaturase [117]. Indeed, BBR inhibited both SREBP1c and SREBP2 expression in hepatocytes, in accordance with an effect on AMPK [88, 115].

PPAR*α* is a major regulator of lipid metabolism in the liver and activation of PPAR*α* promotes uptake, utilization, and catabolism of fatty acids by the regulation of genes involved in fatty acid transport, binding, and activation, as well as peroxisomal and mitochondrial fatty acid *β*-oxidation [118]. Carnitine palmitoyltransferase 1*α* (CPT-1*α*) catalyzes the primary regulatory step in overall mitochondrial fatty acid oxidation [119]. BBR binds directly to the ligand-binding domain of PPAR*α* and upregulates CPT-1*α* gene and protein expression in HepG2 cells and hyperlipidemic rat liver [120]. AMPK and p38 mitogen-activated protein kinase (MAPK) are involved in the activation of PPAR*α* [121, 122]. Expression of c-Jun-N-terminal-kinase (JNK) is negatively correlated with fatty acid utilization, and, in contrast, deficiency of JNK enhances fatty acid utilization [123]. Emerging evidence indicates that whereas JNK1/2 isoforms promote obesity and insulin resistance which are both linked to hypertriglyceridemia and decreased JNK3 activity may protect from excessive adiposity [124], and further p38 MAPK antagonizes JNK [125]. Evidence is emerging that while AMPK plays a central role, BBR regulates lipid metabolism through multiple pathways involving not only APMK but p38 MAPK, JNK, and PPAR*α* [12] as well.

Moreover, BBR inhibits a wide range of intestinal microbes and modulates gut microflora structure and population. This effect results in beneficial gut bacteria such as short-chain fatty acid producers and a decrease in gut* E. Coli* levels in rats fed a high-fat diet [126, 127], which favors body fat oxidation and lowering of blood TAG. Further support is provided by the results of metabolomics analysis, which indicated that BBR treatment resulted in increased pyruvic acid, serotonin, and ketogenic and glycogenic amino acid levels in the serum [127].

## 7. Bioavailability

Although strong cholesterol- and TAG-lowering effects have been demonstrated in numerous studies conducted on animal models and humans, the use of BBR as a drug or dietary supplement is, to date, limited. One of attributable factors is BBR's poor oral bioavailability [128] that has been reported to be less than 1% [58, 59] (Table 3). In rats, the Cmax of BBR in plasma was 11 ng/ml after a single oral administration of 50 mg/kg [57]. In humans, the plasma Cmax of BBR was 0.4 ng/ml after a single oral dose of 400 mg [56]. The poor oral bioavailability of BBR can be attributed to the following aspects: (1) BBR exhibits self-aggregation, which decreases the solubility of BBR in the gastrointestinal tract; (2) BBR has poor permeability across the intestinal mucous membrane; (3) BBR has also been confirmed to be a P-glycoprotein (P-gp) substrate, which limits its transport through the gut wall; and (4) a first-pass effect exists both in the intestine and in the liver [129]. Interestingly, many studies have shown that bioavailability of BBR can be significantly improved by coadministration of BBR with absorption enhancers. For example, coadministration of BBR with sodium caprate increased the oral bioavailability [130]. A similar effect was observed with an amorphous solid preparation of BBR and sodium caprate [62]. Sodium caprate is a medium chain fatty acid that increases intestinal paracellular permeability through enlarging tight junctions, thereby expanding paracellular routes for water-soluble, low lipophilic, and poorly absorbable drugs [131–133] and, in addition, sodium caprate inhibits BBR self-aggregation [130]. Sodium caprate also inhibits the excretion pump function of P-gp [134]. Coadministration of another P-gp inhibitor, herbal polyphenol, demonstrated improvement of oral bioavailability of BBR [50]. Other enhancers include sodium deoxycholate and chitosan [63], which increase BBR's absorption by increasing solubility and intestinal mucous membrane permeability [135].

Rapid biotransformation and metabolism may be other factors that contribute to the low plasma concentrations after BBR supplementation [43, 136]. Besides the three known metabolites, namely, demethyleneberberine-2-O-sulfate (HM1 and RM3), jatrorrhizine-3-O-sulfate (HM5), and thalifendine (RM5), there have been six new metabolites recently identified, which are jatrorrhizine-3-O-*β*-D-glucuronide (HM2), thalifendine-10-O-*β*-D-glucuronide (HM3), berberrubine-9-O-*β*-D-glucuronide (HM4 and RM2), 3,10-demethylpalmatine-10-O-sulfate (HM6 and RM4), columbamin-2-O-*β*-D-glucuronide (HM7), and demethyleneberberine-2,3-di-O-*β*-D-glucuronide (RM1) in human and rat urine samples [137]. This finding also suggests that BBR undergoes similar postabsorption biotransformation and metabolism in the intestine and liver in rats and humans.

Poor absorption in the small intestine is a key factor that presently limits the efficacy of BBR, as such formulators are developing novel formulations aiming to overcome this limitation. For example, a solid dispersion (BPTS-SD) complex composed of a BBR-phospholipid complex (BPC), D-alpha-tocopheryl polyethylene glycol 1000 succinate (TPGS 1000), and SiO2 was prepared and examined. BPC improves liposolubility of BBR, SiO_2_ improves the flowability of BPTS-SD, TPGS 1000 acts as a solid dispersion carrier to improve the dissolution of BPC, and a P-gp inhibitor is included to enhance the intestinal absorption of BBR. The product increased cumulative dissolution rate by 2-3-fold, the absorption of BBR up to 2-fold, and Cmax and AUC_0-∞_ by 2-fold [138]. Other products under development include the use of bioadhesive microspheres, emulsions, microemulsions, and nanoemulsions [68]. Oral bioavailability of BBR was doubled when it was provided in a microemulsion as compared to a standard emulsion formulation [139]. When BBR was delivered using a microemulsion prepared with pharmaceutically acceptable ingredients such as oleic acid, Tween 80, and PEG400, its bioavailability was 6.5 times as compared to that of a BBR tablet suspension [68]. When BBR was provided in bioadhesive microspheres, the bioavailability was enhanced by about 1.5-fold as compared to a commercial tablet form [140]. Significant improvement of oral bioavailability was also observed in both* in vitro* and* in vivo* experiments where BBR was loaded in cremochylomicrons as compared with free BBR [141]. Solid lipid nanoparticles have received great attention in recent years because of their various applications, including formulations to enhance oral drug delivery. A study showed that oral administration of BBR loaded in solid lipid nanoparticles at 100 mg/kg was more potent than the same dose of BBR alone in lowering body weight and fasting blood glucose as well as improving insulin resistance, impaired oral glucose tolerance, and insulin tolerance in* db/db* diabetic mice [57]. With the development and application of emulsions and nanoparticulates, the bioavailability and efficiency of BBR are expected to improve substantially, which will lead in parallel to the reduction of dosages and ultimately to the improvement in acceptance of BBR as a natural cholesterol- and TAG-lowering agent.

## 8. Tolerability and Safety of BBR

The body of evidence derived from animal and human studies support the notion that BBR is generally well tolerated and safe at the doses used for lipid-lowering [74]. BBR's side effect profile was examined in a number of studies and it was found that BBR had mild to moderate effects mostly related to gastrointestinal upset including diarrhea, constipation, and abdominal distension but the incidence and severity of such effects were actually comparable to the control groups [142]. Similar findings were reported in other meta-analyses and systemic reviews of clinical trial data where BBR was tested in groups of patients who were either hyperlipidemic, hypertensive, or diabetic and no serious adverse reactions were reported [74, 78]. A recent publication from an International Lipid Expert Panel has stated that the use of BBR at doses ranging between 500 mg and 1500 mg per day has proved to be effective in lipid-lowering and relatively safe in both primary and secondary prevention [81].

## 9. Conclusion

Numerous studies have been conducted in animal models and humans to demonstrate the cholesterol- and TAG-lowering effects of BBR. Although many different experimental designs have been employed in both the animal and human studies, the majority of these studies consistently show that BBR lowers blood T-C and LDL-C levels while beneficially increasing or having no effect on HDL-C levels. The various mechanisms reported to date mainly center on influencing intestinal cholesterol absorption, LDL-receptor-mediated LDL cholesterol clearance, cholesterol catabolism by conversion to bile acids and subsequent secretion into bile, and free cholesterol secretion into bile. These mechanisms are interregulated and, to date, their relationships and interdependences are not clear, meaning that the observed effects might be a result of actions on multiple processes or regulatory targets. An additional benefit of BBR resides in its TAG-lowering effect. While AMPK appears to play a central role in regulating TAG metabolism by BBR, multiple pathways are reported to be involved including AMPK, p38 MAPK, JNK, and PPAR*α*. As BBR has poor solubility and low oral bioavailability that impact BBR's efficacy, a number of laboratories and research groups have been developing novel oral delivery materials and creating new formulations aimed at improving BBR's bioavailability. Based on the reported data from a number of human studies, oral administration of BBR at a dose range of 0.5–1.5 g/d may be used to lower blood cholesterol, depending on delivery matrix and lipid level of a subject and is considered safe in general; however, caution should always be taken by those who also take other medications. The application of new technology developed to enhance and further improve the oral bioavailability of BBR may result in a significant decrease of the effective oral dose of BBR and increase the acceptance and use of BBR as a functional ingredient in the formulation of future nutraceutical products to control, reduce, or mitigate health risk associated with hyperlipidemia.

## Figures and Tables

**Table 1:** Lipid-lowering effect of BBR in rodent models (rats, mice, and hamsters).

Animal model	Diet	Dose, administration route, time	Effects on blood lipids	Reference
Male SD rats	High-fat and high-cholesterol diet	50, 100, 150 mg/kg·d, i.g., once daily, 8 wk	T-C (−29%, −33%, and −33%), non-HDL-C (−31%, −41%, and −38%), cholesterol absorption rate (−40%, −49%, and −51%) at 50, 100, and 150 mg/kg·d, respectively, no difference among the three doses	[10]

Male Golden Syrian hamsters	High-fat and high-cholesterol diet	46.7 mg/kg·d, i.g., once daily, 120 d	T-C (−19%), LDL-C (−15%), HDL-C (+13%)	[13]

Male C57BL/6J mice	High-fat diet	0.75, 1.5, and 3 mg/kg·d, i.p., once daily, 36 d	T-C (−18.7%, −22.2%, −28%), TAG (−31.2%, −25.2%, −37.8%) at 0.75, 1.5, and 3 mg/kg·d, respectively	[15]

Diabetic male Kunming mice (STZ-induced)	Rodent chow	100 mg/kg·d in *Rhizoma Coptidis* extract, i.g., once daily, 4 wk	T-C (−16%), LDL-C (−20%), TAG (−10%), HDL-C (+9%)	[16]

Diabetic male SD rats (STZ-induced)	High-fat diet	50, 100, and 200 mg/kg·d, i.g., once daily, 8 wk	TC (−7.5%, −44%, −47%), LDL-C (−6.6%, −35%, −19%), TAG (−6.5%, −52%, −44%), HDL-C (+10%, +36%, +29%) at 50, 100 and 200 mg/kg·d, respectively	[17]

Dyslipidemic male Golden Syrian hamsters	High-fat and high-fructose diet for 4 wk and then BBR for 2 wk	150 mg/kg·d, i.g., once daily, 2 wk	LDL-C (−35%), TAG (−47%)	[18]

Male C57BLKS/J-Lepr^*db/db*^ mice	Rodent chow	5 mg/kg·d, i.p., once daily, 3 wk	↓TAG, ↓T-C	[9]

Male SD rats	High-fat diet	200 mg/kg·d, i.g., once daily, 8 wk of dieting and then 16 wk of treatment	↓T-C, ↓LDL-C	[19]

Male SD rats	High-fat diet	200 mg/kg·d, i.g., once daily, 16 wk treatment after 8 wk of dieting	↓T-C, ↓LDL-C	[20]

Female Golden hamsters	High-fat and high-cholesterol diet	50 or 100 mg/kg·d, i.g., twice daily, 14 d of dieting and then 10 d treatment	↓T-C, ↓LDL-C	[7]

Diabetic Golden Syrian hamsters of either sex (STZ-induced)	High-fat and high-cholesterol diet, 4 wk dieting prior to low STZ injection	150 mg/kg·d, i.g., once daily, 9 wk	↓T-C, ↓LDL-C, ↓TAG, ↑HDL-C	[21]

Wistar rats (sex not specified)	High-fat diet	380 mg/kg·d, i.g., once daily, 2 wk treatment after 4 wk dieting	↓TAG	[22]

Hyperhomocysteinemic male SD rats	High-methionine diet	5 mg/kg·d, i.p., once daily, 5 d treatment after 4 wk dieting	↓T-C	[23]

Female C57BL/6 mice	Rodent chow and high-cholesterol diet	10 or 30 mg/kg·d, i.g., once daily, 4 wk rodent chow and then 1 wk high-cholesterol diet	↓TC, ↓TAG, ↓LDL-C	[24]

Female SD rats	Rodent chow	385 mg/kg·d, i.g., once daily, 2 wk	T-C (−9%), TAG (−35%)	[25]

Diabetic male SD rats (STZ-induced)	High-fat diet	100 or 200 mg/kg·d, i.g., once daily, 8 wk	TAG (−30%), TC (−35%) at 100 mg/kg·d	[26]

Diabetic male Wistar rats (STZ-induced)	AIN-93G diet	100 mg/kg·d, i.g., once daily, 7 wk	↓NEFA.	[27]

Diabetic male Wistar rats (STZ-induced)	High-fat and high-sucrose diet	15 or 30 mg/kg·d, i.g., once daily, 6 wk	T-C (−44%), TAG (−42%) at the dose of 30 mg/kg·d	[28]

Male diabetic KKAy mice	High-fat diet	250 /kg·d, i.g., once daily, 4 wk	TC (−42%), TAG (−42%)	[12]

Hyperlipidemic male rats and mice	High-fat and high-cholesterol diet	100 mg/kg·d, i.g., once daily, 30 d in rats and 21 d in mice	In rats, T-C (−27%) and LDL-C (−32%) by compound 1; T-C (−43%) and LDL-C (−49%) by compound 2. In mice, T-C (−17%) and LDL-C (−24%) by compound 1; T-C (−30%) and LDL-C (−39%) by compound 2	[29]

Diabetic male Wistar rats (STZ-induced)	High-fat diet	380 mg/kg·d, i.g., once daily, 4 wk	T-C (−17%), LDL-C (−60%), TAG (−30%), HDL-C (+26%)	[30]

Male SD rats	High-fat and high-cholesterol diet	100 mg/kg·d BBR and 1% plant stanols in diet, 6 wk	T-C (−41%), non-HDL-C (−59%), TAG (−17%)	[11]

Hypercholesterolemic male Golden Syrian hamsters	High-cholesterol diet	1.8 mg/kg·d, i.p., once daily, 24 d	T-C (−32%), LDL-C (−26%) and TAG (−33%) by goldenseal extract; T-C (−30%), LDL-C (−27%) and TAG (−34%) by BBR	[31]

Diabetic male Wistar rats (alloxan-induced)	High-cholesterol diet	100 or 200 mg/kg·d, i.g., once daily, 21 d	T-C (−14% or −20%); LDL-C (−37% or 44%) at 100 mg/kg·d or 200 mg/kg·d	[32]

Hypercholesterolemic male Golden Syrian hamsters	High-fat and high-cholesterol diet	100 mg/kg·d, i.g., once daily, 10 d treatment after 2-wk dieting	T-C (−27%), LDL-C (−39%)	[33]

Diabetic male Wistar rats (STZ-induced)	Regular chow	187.5 or 562.5 mg/kg·d, i.g. once daily, 8 wk	T-C (−18%), TAG (−67%) and HDL-C (+36%) at 187.5 mg/kg·d; T-C (−18%), TAG (−66%) and HDL-C (+27%) at 562.5 mg/kg·d	[34]

Male SD rats	High-fat and high-cholesterol diet	300 mg/kg·d, 60 mg/kg·d, i.g., once daily, 12 wk	↓T-C, ↓LDL-C, ↑HDL-C	[35]

*Note*. If the results of a study are presented in figures, no percent reductions are available.

**Table 2:** Effect of BBR on blood lipid profiles in humans.

Subject	Treatment	Dose, frequency, time	Effects	Reference
Anovulatory Chinese women with polycystic ovary syndrome	BBR, *n* = 98 (69 normal weight and 29 overweight/obese)	1.2 g/d, thrice daily, 4 months	T-C (−17%), LDL-C (−12%), and TAG (−37%)	[36]

Hyperlipidemic subjects	BBR-containing nutraceutical^$^, *n* = 30 (15/15, M/F); Placebo, *n* = 9 (3/6, M/F)	0.2 g/d, once daily, 12 wk	Non-HDL-C (−15%), LDL-C (−19%)	[37]

Moderately hypercholesterolemic subjects	AP-1, *n* = 51 (18/33, M/F); Placebo, *n* = 51 (14/37, M/F)	500 mg/d, once a day, 12 wk	TC (−5%), LDL-C (−7.8%)	[38]

Patients with type-2 diabetes	BBR, *n* = 37 (17/20, M/F); compared to baseline	0.9 g/d, thrice daily, 3 months	T-C (−11%), LDL-C (−16%), TAG (−21%)	[39]

Hypercholesterolemic patients	BBR, *n* = 63 (35/28, M/F); Placebo, *n* = 28 (17/11, M/F)	1 g/d, twice daily, 3 months	T-C (−29%), LDL-C (−25%), TAG (−35%)	[7]

Hypercholesterolemic patients	BBR, *n* = 24; compared to baseline (no sex ratio provided)	1 g/d, twice daily, 2 months	TC (−21.8%), LDL-C (−23.8%), TAG (−22.1%)	[40]

Patients with type-2 diabetes	BBR, *n* = 15; compared to baseline (no sex ratio provided)	1.5 g/d, thrice daily, 13 wk	TC (−13%), TAG (−21%)	[41]

Patients with type-2 diabetes	BBR, *n* = 50 (27/23, M/F); compared to baseline	1 g/d, twice daily, 2 months	TAG (−18%).	[42]

Patients with type-2 diabetes and dyslipidemia	BBR, *n* = 58 (30/28, M/F); Placebo, *n* = 52 (31/21, M/F)	1 g/d, twice daily, 3 months	T-C (−18%), LDL-C (−21%), TAG (−36%)	[43]

Caucasians with low cardiovascular risk	BBR, *n* = 71 (35/36, M/F); Placebo, *n* = 70 (35/35, M/F)	1 g/d, twice daily, 3 months	T-C (−11.6%), LDL-C (−16.4%), TAG (−21.2%), HDL-C (+9.1%)	[44]

Dyslipidemic patients	AP-1^*δ*^, *n* = 933 (416/518, M/F); Placebo, *n* = 818 (384/434, M/F)	500 mg/d, once daily, 16 wk	T-C (−10%), LDL-C (−13%), TAG (−7%), HDL-C (+8%)	[45]

Patients with metabolic syndrome	BBR, *n* = 12; Placebo, *n* = 12 (no sex ratio provided)	1.5 g/d, thrice daily, for 3 months	TAG (−42%)	[46]

Patients on hormone-therapy after breast cancer	AP-1, *n* = 21; compared to baseline	500 mg/d, once daily, 3 months	T-C (−15%), LDL-C (−19%), TAG (−37%)	[47]

Hypercholesterolemic subjects	AP-1, *n* = 152 (62/90, M/F); compared to baseline	500 mg/d, once daily, 6 months	TC (−24%), LDL-C (−32%), non-HDL-C (−30%), TAG (−20%)	[48]

Moderate dyslipidemic subjects	BBR, *n* = 20 (8/12, M/F); AP-1, *n* = 20 (8/12, M/F)	500 mg/d, once daily, 4 wk	T-C (−16%), LDL-C (−20%), TAG (−22%), HDL-C (+7%); AP-1 and BBR did not differ	[49]

Patients with type-2 diabetes	Berberol^#^, *n* = 22 (17/5, M/F); compared to baseline	500 mg/d, once daily, 90 d	T-C (−21%), LDL-C (−19%), TAG (−44%)	[50]

Obese Caucasians	BBR, *n* = 7, compared to baseline (no sex ratio provided)	1.5 g/d, thrice daily, 12 wk	T-C (−12%), TAG (−23%)	[25]

Patients with metabolic syndrome	AP-1, *n* = 29 (20/9, M/F); Placebo, *n* = 30 (18/12, M/F)	500 mg/d, once daily, 18 wk	T-C (−15%), LDL-C (−23%)	[51]

Menopausal women with moderate dyslipidemia	BBR + Isoflavones, *n* = 60; compared to baseline	Isoflavones and BBR combination, 12 wk	T-C (−14%), LDL-C (−12%), TAG (−19%)	[52]

Elderly (>75 yr) hypercholesterolemic patients	AP-1, *n* = 40 (21/19, M/F); Placebo, *n* = 40 (20/20, M/F)	500 mg/d, once daily, 12 months	T-C (−20%), LDL-C (−31%)	[53]

Patients with polycystic ovary syndrome and insulin resistance	BBR. *n* = 31; compared to baseline	1.5 g/d, thrice daily, 3 months	T-C (−17%), LDL-C (−14%), TAG (−17%), HDL-C (+12%)	[54]

Hypercholesterolemic patients	AP-1, *n* = 25 (13/12, M/F); Placebo, *n* = 25 (13/12, M/F)	500 mg/d, once daily, 6 wk	T-C (−17%), LDL-C (−23%)	[55]

^*δ*^AP-1: 1 tablet contains red yeast rice extract 200 mg (equivalent to 3 mg monacolins), policosanol 10 mg, BBR 500 mg, folic acid 0.2 mg, CoQ_10_ 2 mg, and astaxanthin 0.5 mg. ^#^Each tablet contains 558 mg of *B. aristata* extract titered as 85% of BBR and 105 mg of *S. marianum* extract titered as >60% of flavonol lignans. ^$^BBR-containing nutraceutical: BBR 200 mg, monacolin K 3 mg, chitosan 10 mg, and CoQ_10_ 10 mg.

**Table 3:** Bioavailability of BBR in animals and humans.

Subject/research model	Diet	Dose, administration route, treatment time	Bioavailability^*∗*^	Reference
Type-2 diabetic patients	No diet restriction	Berberol, each containing 558 mg of *B. aristata* extract titered as 85% BBR and 105 mg of *S. marianum* extract titered as >60% flavonolignans, 2 tablets/d, once daily, for 90 d	Low oral bioavailability of BBR can be overcome by P-glycoprotein inhibitors like herbal polyphenol. *S. marianum* extract	[50]

Healthy subjects	No diet restriction	400 mg, once i.g.	*C*_max_ = 0.44 ng/mL, *T*_1/2_ = 29 hr, *T*_max_ = 10 hr, AUC_0–96_ = 7.8 (hr·ng/ml), AUC_0–*∞*_ = 9.2 (hr·ng/ml)	[56]

Diabetic Kunming mice (STZ-induced)	Rodent chow	100 mg/kg, once i.g. in Rhizoma Coptidis extract and a classical Chinese prescription, Jiao-Tai-Wan	Addition of *Cinnamomum cassia* increased bioavailability of BBR	[16]

SD rats	Diet not specified	50 mg/kg, once i.g., free BBR or BBR loaded in solid lipid nanoparticles	*C*max = 11.1 ng/mL, *T*_1/2_ = 9.2 hr, *T*max = 2.3 hr, AUC_0–*∞*_ = 86.5 (ug·hr/L) for free BBR *C*max = 44.6 ng/mL, *T*_1/2_ = 11.5 hr, *T*max = 0.38 hr, AUC_0–*∞*_ = 179.4 (ug.hr/L) for BBR loaded in solid lipid nanoparticles	[57]

Wistar rats	Regular diet	40 mg/kg, once i.g.	BBR absorption rate in jejunum was 19.1%, 26.5%, 26.8%, and 33.6% at 10, 20, 40, and 60 min, respectively; AUC_0–limit_ = 37, 1879, 811, 1763, and 356 (ng·hr/ml) for BBR and its metabolites M1, M2, M3, and M4	[58]

SD rats	Regular diet	100 mg/kg, once i.g.	Absolute oral bioavailability was 0.36%	[59]

SD rats,	Regular diet	100 mg/kg, once i.g. in spray-dried mucoadhesive microparticle formulations (BBR-SD)	Increased *C*_max_ by 3.5-fold and AUC by 7-fold. *C*_max_ = 147 ng/ml, *T*_max_ = 1.4 hr, AUC_0–*∞*_ = 819 ng·hr/ml, and *T*_1/2_ = 5.95 hr for free BBR, and 509 ng/ml, 2.86 hr, 5724 ng·hr/ml, and 15 hr for BBR-SD	[60]

Rats with inflammatory bowel disease	Regular diet	5 mg/kg in self-nanoemulsifying drug delivery system, once daily, i.g., 7 d	Improved solubility and therapeutic efficacy in either liquid or solid form of self- nanoemulsifying drug delivery system	[61]

SD rats	Regular diet	100 mg/kg in Huang-Gui solid dispersion, once i.g.	Oral bioavailability was increased by 5-fold	[62]

Wistar rats	Regular diet	100 mg/kg in sodium caprate, or sodium deoxycholate, once i.g.	AUC was increased 41-fold by sodium caprate and 35-fold by sodium deoxycholate.	[63]

SD rats	Regular diet	25 mg/kg in self-emulsifying drug delivery system, once i.g.	Increased peak plasma concentration and AUC (0–12 hr) by 160% and 150%, respectively, and relative bioavailability ~2.4-fold	[64]

Wistar rats	Regular diet	100 mg/kg in chitosan hydrochloride solution, once i.g.	Oral bioavailability was increased by 2.5-fold	[65]

Kunming mice	Regular diet	100 mg/kg in anhydrous reverse micelle delivery system, once i.g.	Enhanced oral bioavailability 2.4-fold	[66]

Wistar rats	Regular diet	100 mg/kg in D-alpha-tocopheryl polyethylene glycol 1000 succinate (TPGS), once i.g.	TPGS at a concentration of 2.5% increased peak serum concentration and AUC of BBR by 3-fold and 2-fold, respectively	[67]

SD rats	Regular diet	50 mg/kg in microemulsion, once i.g.	Increased oral bioavailability 6.5-fold	[68]

^*∗*^AUC: area under the curve of blood BBR concentration in a pharmacokinetic study.
